# Distant survival for patients undergoing surgery using volatile versus IV anesthesia for hepatocellular carcinoma with portal vein tumor thrombus: a retrospective study

**DOI:** 10.1186/s12871-020-01111-w

**Published:** 2020-09-14

**Authors:** Xiao-Yan Meng, Xiu-Ping Zhang, Zhe Sun, Hong-Qian Wang, Wei-Feng Yu

**Affiliations:** 1 Department of Anesthesiology, Eastern Hepatobiliary Surgery Hospital, the Second Military Medical University, 225 Changhai Road, Shanghai, China; 2 Department of Anesthesiology, Ren Ji Hospital, School of Medicine, Shanghai Jiao Tong University, 160 Pudian Road, Shanghai, China; 3 Department of Hepatic Surgery, Eastern Hepatobiliary Surgery Hospital, the Second Military Medical University, 225 Changhai Road, Shanghai, China

**Keywords:** Hepatocellular carcinoma, Portal vein tumor Thrombus, Volatile inhalational anesthesia, Total IV anesthesia

## Abstract

**Background:**

Whether anesthesia type is associated with the surgical outcome of Hepatocellular carcinoma (HCC) patients with portal vein tumor thrombus (PVTT) remains to be determined. This study aims to investigate the impact of volatile inhalational anesthesia (INHA) versus total IV anesthesia (TIVA) on the survival outcomes in HCC patients with PVTT.

**Methods:**

A cohort of in-patients whom were diagnosed of HCC with PVTT in Eastern Hepatobiliary Surgery Hospital, Shanghai, China, from January 1, 2008 to December 24, 2012 were identified. Surgical patients receiving the INHA and TIVA were screened out. The overall survival (OS), recurrence-free survival (RFS) and several postoperative adverse events were compared according to anesthesia types.

**Results:**

A total of 1513 patients were included in this study. After exclusions are applied, 263 patients remain in the INHA group and 208 in the TIVA group. Patients receiving INHA have a lower 5-year overall survival rate than that of patients receiving TIVA [12.6% (95% CI, 9.0 to 17.3) vs. 17.7% (95% CI, 11.3 to 20.8), *P* = 0.024]. Results of multivariable Cox-regression analysis also identify that INHA anesthesia is significantly associated with mortality and cancer recurrence after surgery compare to TIVA, with HR (95%CI) of 1.303 (1.065, 1.595) and 1.265 (1.040, 1.539), respectively. Subgroup analysis suggested that in more severe cancer patients, the worse outcome related to INHA might be more significant.

**Conclusion:**

This retrospective analysis identifies that TIVA is associated with better outcomes compared with INHA. Future prospective studies clinical and translational studies are required to verify this difference and investigate underlying pathophysiology.

## Background

Volatile inhalational (INHA) and IV anesthesia (TIVA) are two methods commonly used in general anesthesia maintenance. Currently, several researches reported that INHA was associated with worse postoperative outcomes compare to INHA in certain types of cancers. Dr. Wigmore et al. [1] did a retrospective analysis which firstly compared long-term survival in more than 7000 patients undergoing elective cancer surgeries, and reported that mortality of patients accepted INHA is approximately 50% greater than those accepted TIVA. Since then, more studies reported similar results in different cancers [2]. Besides from these clinical evidences, animal researches also reported that administration of volatile inhalational agents was associated with up-regulation of tumorigenic growth factors including hypoxia-inducible factors (HIFs) and insulin-like growth factor (IGF) [3, 4], which are highly associated with progression angiogenesis and cell proliferation in tumor.

Although the underlying mechanism remains unclear, these results have drawn due attention that anesthesia technique might be an independent risk factor for postoperative outcomes of most cancers, including liver cancer. Of note, previous studies also reported that the MAC of sevoflurane is lower in patients with end-stage liver cancer [5]. Thus, we hypothesize that INHA might be associated with lower 5-year overall survival (OS) compared with TIVA in hepatocellular carcinoma (HCC) patients with portal vein tumor thrombus (PVTT), an end-stage liver cancer with a high recurrence rate and reduced median survival time (MST) [6–9], in considering that in these end stage cancer patients, even subtle differences in medication might lead to significant effects on long-term outcome.

## Methods

### Study design

We retrospectively identified all patients who underwent aggressive surgical liver resection for selected HCC patients with PVTT at Eastern Hepatobiliary Surgery Hospital from January 1, 2008 to December 24, 2012. Exclusion criteria including: (1) no surgical treatment performed; (2) received mixed inhalational and intravenous anesthesia; (3) received additional procedures with different anesthesia or for other diseases afterwards; (4) received extra sedation in ICU or in general ward after surgery; (5) less than 18 years old; (6) had an urgent or emergence surgery and (7) incomplete follow-up data. The research was approved by the Ethics Committee of the Eastern Hepatobiliary Surgical Hospital of China. Written informed consents to record clinical follow up data were obtained from participants or their surrogates during hospitalization.

Baseline data retrospectively extracted including anesthetic technique, year of surgery, age at the time of surgery, sex, American Society of anesthesiologists’ (ASA) physical status classification, pre-existing diagnosis of diabetes or hypertension, HBV surface antigen (HBsAg) and HCV anti-body (HCV-ab). Data related to patients’ preoperative liver function, cytonecrosis and cancer statue were also documented, including Child-Pugh score, alpha-fetoprotein (AFP), type of PVTT, tumor diameter as well as alanine aminotransferase (ALT) and aspartate aminotransferase (AST) levels.

### Outcomes

The primary outcome was 5-year OS. Secondary outcomes were (1) recurrence-free survival (RFS); (2) 30-day mortality; (3) a set of major adverse cardiac events (MACE) that included myocardial infarction (MI), cardiac arrest, or newly diagnosed malignant arrhythmia; (4) multiple organ dysfunction (MOD) primarily induced of acute hepatic failure postoperatively; (5) blood loss and blood transfusion; (6) hospital length of stay (7) postoperative ALT and AST were also recorded.

### Anesthesia techniques

Patients were divided based on INHA or TIVA they received for maintenance of anesthesia. Patients in the TIVA group received continuous infusions of propofol, and those in the INHA group received sevoflurane. Supplementary opioid for maintaining were used at the discretion of the anesthetist in all patients, including sufentanil and/or remifentanil, with the highest dose no more than 50 mg and 2 mg, respectively. No other sedative-hypnotic drugs were used during maintenance.

Type of anesthesia was according to the anesthetist’s decision, mainly depending on their preference and proficiency of the anesthesia technique. Details of the surgical process as previously described [10].

### Statistical analyses

The Kaplan–Meier method was used to calculate the overall survival and recurrence-free survival of patients from the date of surgery to the date of events. A univariable Cox regression analysis was applied, and for variables with P less than 0.1 were then included into the multivariable model to identify risk factors.

Secondary outcomes were compared using chi-square or Mann-Whitney-Wilcoxon tests as appropriate. Missing values (all less than 5%) were filled by the average value of the variable. Significant difference defined as *P* < 0.05 in all analysis (SPSS version 22.0; IBM Inc., USA).

## Results

### Baseline characters and survival for all patients

**Fig. 1:**
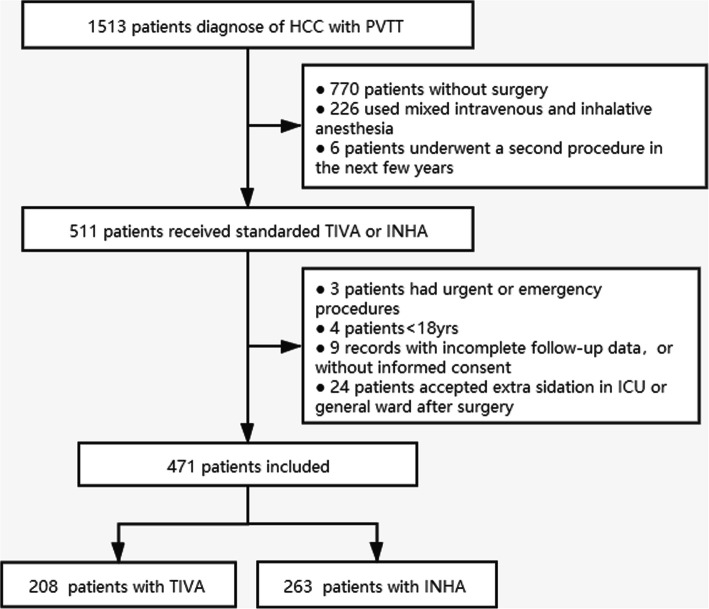
Flow diagram detailing the selection of patients included in the retrospective analysis. INHA = volatile inhalational; TIVA = total IV anesthesia

A total of 1523 patients whom are diagnosed of HCC with PVTT are delivered in the study period. After exclusions applied, 471 patients are included in the analysis, with 263 patients in the INHA group and 208 in the TIVA (Fig. 1). The mean age is 48.6 years old; The majority of patients are male (90.6%), had a grade of ASA II (88.3%) and Child-Pugh A (88.4%); 410 (87.0%) of patients have large hepatocellular carcinoma (> 10 cm).; 408 (86.6%) are identified with HBsAg−+, including 4 with both HBsAg− + and HCV-ab+. Only 4 patients are identified with HCV-ab− + alone, which is not enough for effective analysis. Five-year survival rate for all patients is 14.8% (95% CI, 11.3 to 17.6), with median survival time of 9.0 month (95% CI, 7.9 to 10.0). The patient characteristics in two groups are described in Table 1.

**Table 1:** Patient baseline characters

Variables	TIVA (*N* = 208)	INHA (*N* = 263)	*P* value
	N (%)	N (%)	
Sex (male)	188 (90.4)	239 (90.9)	0.856
HBsAg − +^a^	182 (87.5)	226 (85.9)	0.619
ASA			
II	186 (89.4)	229 (87.1)	0.191
III	22 (10.6)	30 (11.4)	
IV	0 (0)	4 (1.5)	
Child-Pugh			
A	184 (88.5)	233 (88.6)	0.662
B	22 (10.6)	25 (9.5)	
C	2 (0.4)	5 (1.1)	
AFP (ug/L)			
< 25	30 (14.4)	54 (20.5)	0.109
25–399	39 (18.8)	45 (17.1)	
400–999	12 (5.8)	25 (9.5)	
≥ 1000	127 (61.1)	139 (52.9)	
Tumor Diameter (cm)			
< 5	1 (0.4)	2 (0.8)	0.546
5–9.9	22 (10.6)	36 (13.7)	
≥ 10	185 (88.9)	225 (85.6)	
PVTT			
1	30 (14.4)	40 (15.3)	0.716
2	131 (63.0)	163 (62.0)	
3	47 (22.1)	60 (22.8)	
Year of surgery			
2008	70 (33.7)	79 (30.0)	0.882
2009	33 (15.9)	42 (16.0)	
2010	31 (14.9)	46 (17.5)	
2011	31 (14.9)	37 (14.1)	
2012	43 (20.7)	59 (22.4)	
	Mean (SD)	Mean (SD)	
Age (yr)	48.0 (10.94)	49.0 (9.73)	0.078
	Median (IQR)	Median (IQR)	
WBC (10^9^/L)	5.5 (4.2, 7.3)	5.4 (4.3, 7.1)	0.06
ALT (U/L)	47.8 (31.0, 66.0)	45.0 (29.1, 67.3)	0.903
AST (U/L)	49.0 (37.0, 69.0)	52.0 (35.0, 71.0)	0.820

*ASA* American Society of Anesthesiologists, *PVTT* Portal vein tumor thrombus, *INHA* Volatile inhalational anesthesia, *TIVA* Total IV anesthesia, *WBC* White blood cells, *NLR* Neutrophil–lymphocytes ratio

^a^ Including 4 patients with HBsAg− + and anti-HCV − +

### Five-year OS and RFS

**Fig. 2:**
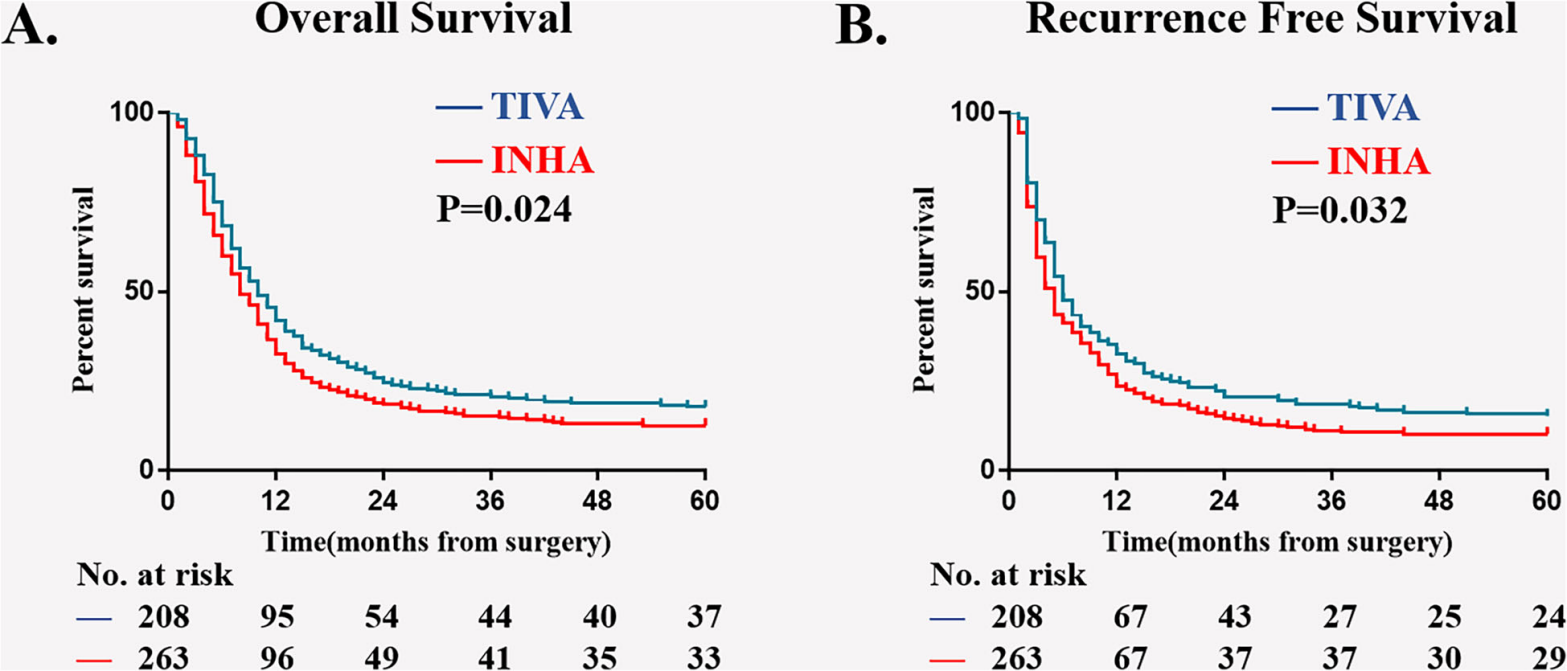
Kaplan–Meier survival curves from the date of surgery by anesthesia type for (**a**) overall survival in patients before matching (*P* = 0.007), (**b**) overall survival in patients after matching (*P* = 0.044), (**c**) recurrence-free survival in patients before matching (*P* = 0.020), (**d**) recurrence-free survival in patients after matching (*P* = 0.081). INHA = volatile inhalational; TIVA = total IV anesthesia

Results of Kaplan–Meier survival analysis show that, compare with TIVA, INHA is associated with a worse 5-year OS rate [17.7% (95% CI, 11.3 to 20.8 VS. 12.6% (95% CI, 9.0 to 17.3)); *P* = 0.024, Fig. 2a], as well as a worse 5-year RFS rate[15.4% (95% CI, 12.6 to 18.1) VS. 11.7% (95% CI, 9.7 to 13.8); *P* = 0.032, Fig. 2b]. On univariable analysis, 6 potential risk factors have *P* < 0.1 are included in multivariable model (Supplementary table 1). Results of multivariable analysis also suggest that INHA is an independent risk factor for mortality [HR (95%CI), 1.303 (1.065, 1.595)] and cancer recurrence [HR (95% CI), 1.265 (1.040, 1.539); Table 2] in 5 years after surgery.

**Table 2:** Cox proportional hazard regression analyses: multivariable model for overall survival and recurrence-free survival

Variables	Overall Survival		Recurrence-Free Survival	
	HR (95% CI)	*P* Value	HR (95% CI)	*P* Value
Anesthesia type (INHA/TIVA)	1.303 (1.065, 1.595)	0.010	1.265 (1.040, 1.539)	0.019
Child-Pugh	1.897 (1.491, 2.414)	0.000	1.653(1.297, 2.105)	0.000
AFP (ug/L)	1.099 (1.010, 1.194)	0.027	1.071 (0.989, 1.160)	0.093
Tumor Diameter (cm)	1.606 (1.183, 2.181)	0.002	1.492(1.123, 1.983)	0.006
PVTT	1.160 (0.989, 1.360)	0.068	–	–

*ASA* American Society of Anesthesiologists, *PVTT* Portal vein tumor thrombus, *INHA* Volatile inhalational, *TIVA* Total IV anesthesia, *NLR* Neutrophil–lymphocytes ratio

### Other secondary outcomes

Other outcomes including 30-day mortality rate, postoperative MACE and MOF rate, as well as blood loss, blood transfusion and length of stay in hospital are similar in both groups (Table 3). Postoperative serum biomarker of ALT and AST are compared (with incomplete data), the results suggest a minor liver cytonecrosis of TIVA after surgery (Supplementary Figure 1).

**Table 3:** Adverse outcomes

	TIVA	INHA	*P* Value
	N (%)	N (%)	
Dichotomous Outcomes			
30-day Mortality	4 (2.1)	11 (4.7)	0.106
MACE	4 (2.1)	11 (4.7)	0.106
MOD	6 (3.3)	9 (3.9)	0.797
Blood Transfusion	80 (43.5)	78 (33.5)	0.189
	Median (IQR)	Median (IQR)	
Continuous Outcomes			
Blood Loss	400 (245,800)	400 (300,800)	0.301
Length of Stay (days)	15 (13,20)	16 (13,20)	0.920

*IQR* Interquartile range, *MACE* Major adverse cardiac events, *MOF* Multiple organ failure, *RR* Risk ratio

### Subgroup analysis

**Fig. 3:**
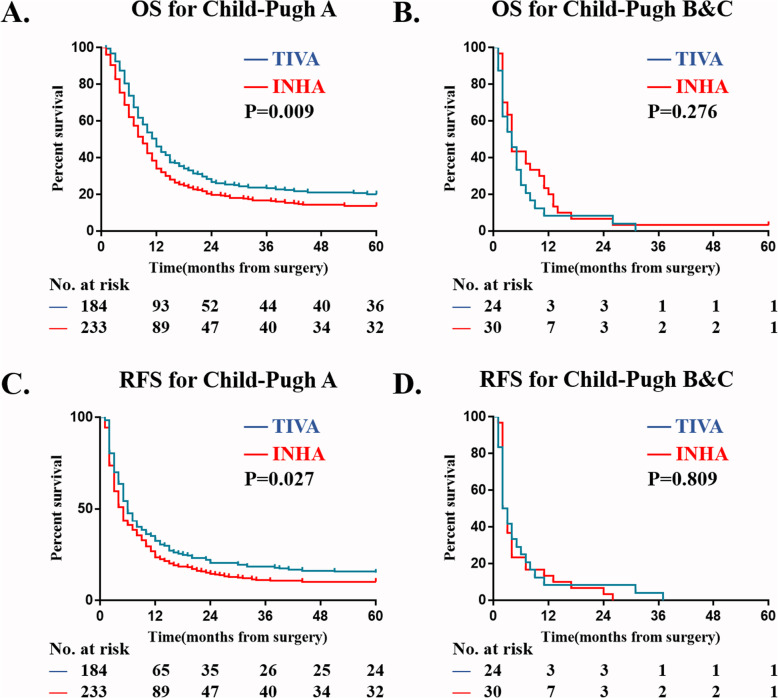
Subgroup Kaplan–Meier survival analysis for anesthesia type on (**a**) OS in Child-Pugh A; (**b**) OS in Child-Pugh B&C; (**c**) RFS in Child-Pugh A; (**d**) RFS in Child-Pugh B&C

**Fig. 4:**
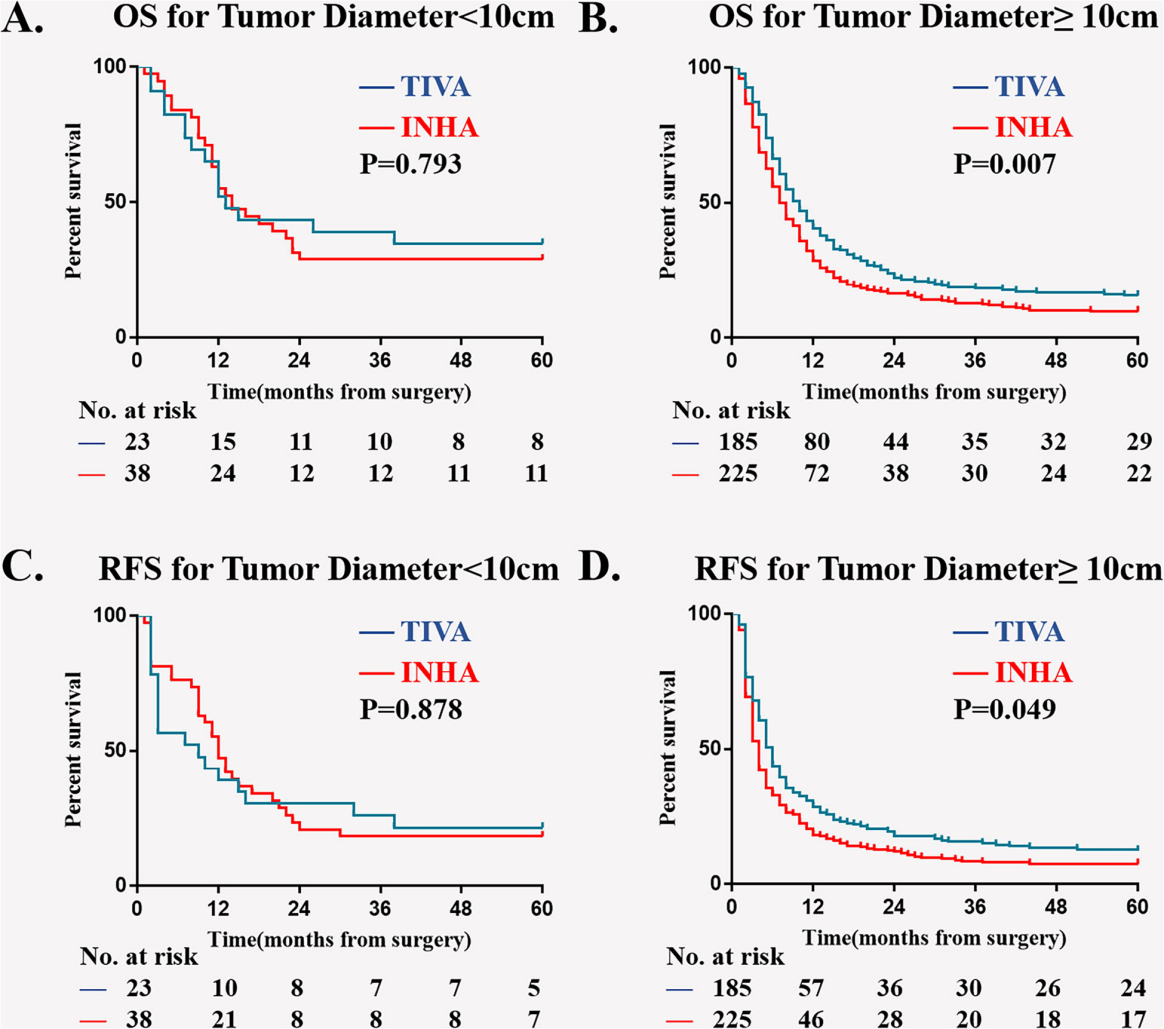
Subgroup Kaplan–Meier survival analysis for anesthesia type on (**a**) OS in tumor diameter < 10 cm; (**b**) OS in tumor diameter ≥ 10 cm; (**c**) RFS in tumor diameter < 10 cm; (**d**) RFS in tumor diameter ≥ 10 cm

**Fig. 5:**
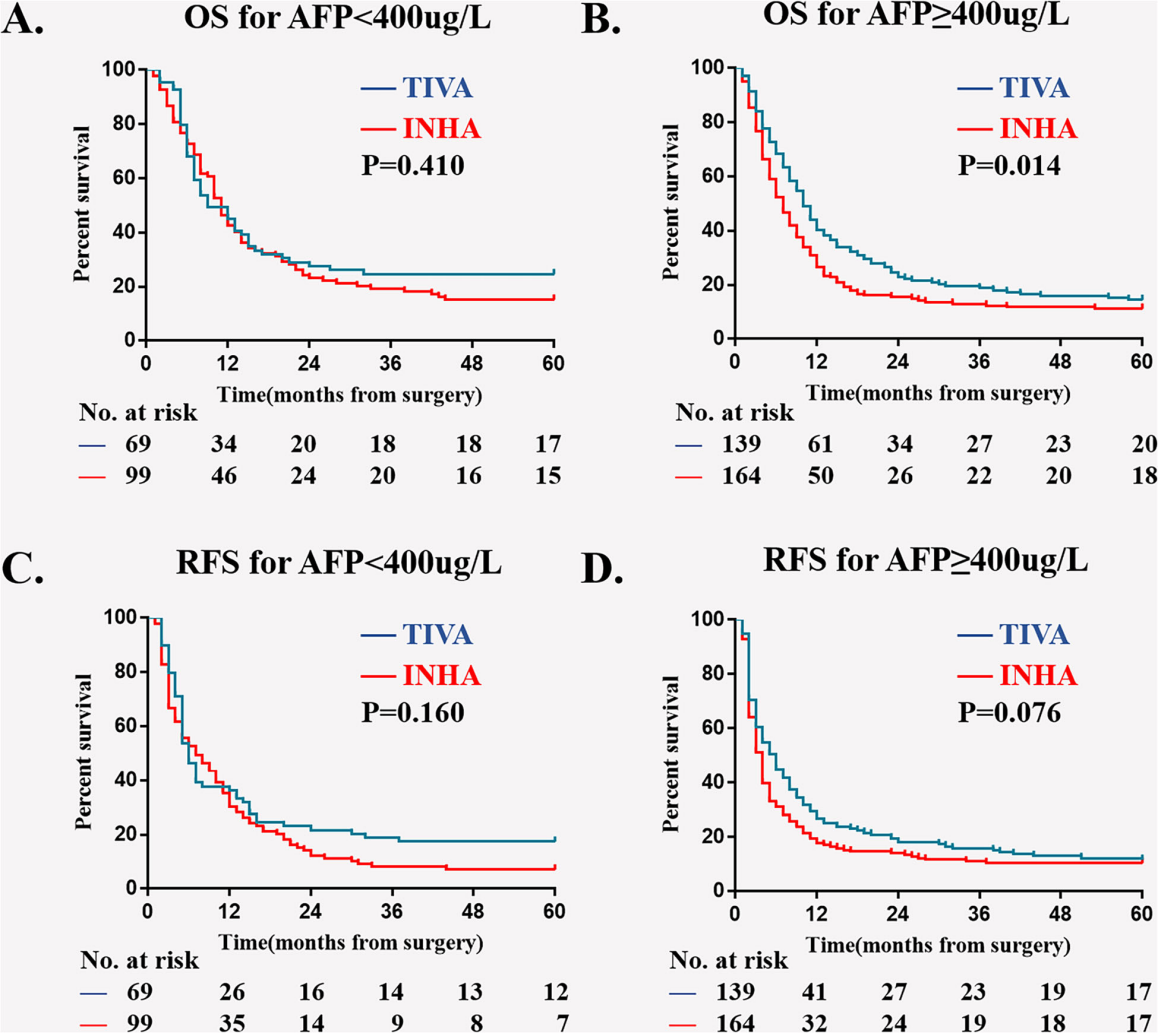
Subgroup Kaplan–Meier survival analysis for anesthesia type on (**a**) OS in AFP < 400μg/L; (**b**) OS in AFP ≥ 400μg/L; (**c**) RFS in AFP < 400μg/L; (**d**) RFS in AFP ≥ 400μg/L

**Fig. 6:**
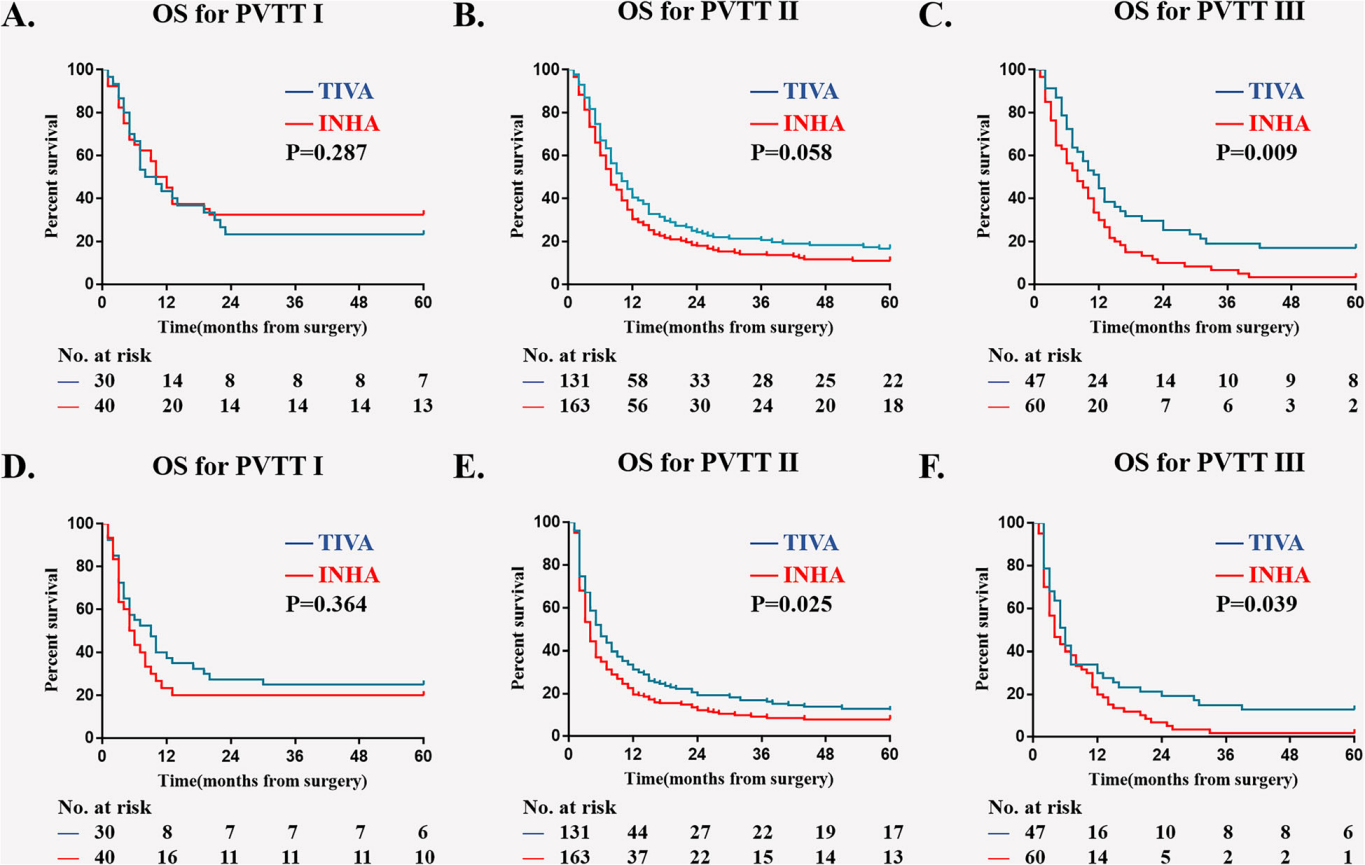
Subgroup Kaplan–Meier survival analysis for anesthesia type on (**a**) OS in PVTT typeI; (**b**) OS in PVTT typeII; (**c**) OS in PVTT typeIII; (**d**) RFS in PVTT typeI; (**e**) RFS in PVTT typeII; (**f**) RFS in PVTT typeIII

In multivariable model, four more variables are screened out as independent risk factors for 5-year OS and RFS: Child-Pugh, AFP level, diameter of hepatocellular carcinoma and PVTT type. We then did a subgroup Kaplan–Meier survival analysis to estimate the association of anesthesia type on postoperative OS and RFS in different sub-variable groups, there were Child-Pugh A; Child-Pugh B&C; tumor diameter < 10 cm; tumor diameter ≥ 10 cm; AFP < 400μg/L; AFP ≥ 400μg/L; PVTT typeI; PVTT typeII; PVTT typeIII (Figs. 3, 4, 5, and 6). The results suggest that INHA was associated with significant lower OS and RFS rate compare with TIVA in several sub-variable groups indicating more severe liver cancer status, including tumor diameter ≥ 10 cm (Fig. 4b, d); AFP ≥ 400μg/L (Fig. 5b, d); PVTT typeIIand PVTT typeIII (Fig. 6c, e, f).

## Discussion

This retrospective analysis evaluates long-term OS, RFS and several short-term postoperative adverse events in 1513 HCC patients with PVTT receiving INHA and TIVA. We identify that patients receiving INHA using sevoflurane had a lower 5-year OS and RFS rate than that of patients receiving TIVA using propofol. On multivariable Cox regression analysis, we identify that INHA is an independent risk factor for mortality and cancer recurrence in 5-year after surgery. In subgroup analysis, our results suggest that patients accepted INHA, compare with those who accepted TIVA, have worse survival rates when there are in severe liver cancer status. No significant differences in postoperative adverse events and 30-day mortality are found between the two groups in this study.

### Clinical evidence of anesthesia type on surgical outcomes

Recently, several clinical studies have been investigated for anesthesia type on postoperative outcomes in elective cancer patients. Enlund et al. [6] did a retrospective analysis based on 2838 patients with breast, rectal, and colon cancer, they reported that the overall survival for patients receiving propofol anesthesia is 4.7% higher at 1-yr and 5.6% higher at 5-yr than those receiving sevoflurane. But after balance for confounders, this differences are not significant. In another study, Wigmore et al. [1] analyzed 7030 patients who underwent elective cancer surgery over a 3-yr period, They reported a worse outcome in patients receiving volatile anesthesia, with a HR of 1.46 (95% CI, 1.29 to 1.66) for death, compare with TIVA. In addition, Yan et al. [7]. designed a randomized controlled trail in 80 breast cancer patients, they reported that the total intravenous anesthesia can inhibit the release of vascular endothelial growth factor C (VEGF-C) in breast surgery, yet with no significant benefice in the short-term recurrence rate of breast cancer. Importantly, a recent meta-analysis with 9 retrospective studies and 1 RCT concluded that, the use of TIVA was associated with improved RFS in all cancer types and improved OS in several certain types of cancers [2]. These studies have achieved consistent results that TIVA anesthesia has a better long-term prognosis for patients undergoing tumor resection compared with INHA. However, the population these studies enrolled varies a lot. Yet meaningful conclusions on whether TIVA is superior to INHA for all cancer patients or just for certain types of cancer are difficult to define. To this end, the current study sort to compare the long-term survival rate in TIVA and INHA in HCC patients with PVTT.

### Laboratory evidence of anesthetics on tumors metastasis and recurrence

Over the years, numerous animal and laboratory studies have been investigated for the mechanism of anesthetic agents on primary tumors metastasis and recurrence. Cellular immune system and tumor proliferation-associated factors are considered to play a key role in it. For instance, propofol has been demonstrated to have a preservation effective on T lymphocyte activity and Th1 cytokine secretion, or even inhibits tumor growth in animal model [8, 9, 11]. Of note, T lymphocytes and NK cells are two major cytotoxic effector cells that participate in cell-mediated immune responses. Meanwhile, researches also proved that sevoflurane could inhibit primary leukocyte integrin lymphocyte function and could induced lymphocyte apoptosis through downregulation of Lymphocyte Function-associated Antigen 1 (LFA-1), thus promoting tumor recurrence and metastasis [12]. Moreover, studies both in vivo [13] and in patients undergoing breast cancer surgery [14] have reported an inhibitory effect of anesthetic agents on natural killer cell function, and further promotes tumor recurrence. This inhibitory effect is probably related to the dysfunction in CD16 cell and CD107α NK receptor after exposure to sevoflurane [15]. More recently, Bellanti et al. [16] demonstrated that propofol, but not sevoflurane, prevents mitochondrial dysfunction and oxidative stress by limiting hypoxia-inducible factor 1 alpha (HIF-1α) activation in hepatic ischemia/reperfusion injury. According to their description, this change could be beneficial for liver function, as HIF-1α governs the transcription of genes controlling proliferation and metastasis of tumor cells [17, 18]. Additionally, previous researches already demonstrate that isoflurane administration could result in an up-regulation of HIF-1α in tumor [19]. However, there have no solid evidence to prove those theory in human body, while the molecular mechanisms of the different outcomes of the two anesthetic methods remains to be determined yet.

### HCC with PVTT

HCC ranges as the fifth most common malignancy tumor [20]. Indeed, even worse prognosis is reported in HCC patients with PVTT, with a reported rate of 20% and a reduced median survival time (MST) of around 2–4 months compared to HCC patients without PVTT [21–24]. According to the Asia-Pacific guideline, surgery is recommended as one of the beneficial multidisciplinary treatments for PVTT. Moreover, aggressive surgical resection is associated with a longer survival outcome, and even provide chances for complete cure with type I and II PVTT [10, 25]. Meanwhile, recent studies reported that under advanced perioperative management and skilled surgical operation, the in-hospital mortality of HCC patients with PVTT arrives an acceptable rate ranging from 3.7 to 10% [26, 27]. However, the knowledge about risk factors for postoperative mortality, cancer recurrence and other side events of HCC patients with PVTT still remains insufficient. Our result provides with extra evidence that anesthesia type might be a risk factor for surgical outcomes of HCC patients with PVTT.

### Limitations

Several methodological discrepancies and limitations of this study should be discussed. First, there might be inclusion bias exist in our cohort, as more than a thousand patients were excluded with only 471 enrolled. Meanwhile, the majority of included patients are male, with ASA score of II, Child-Pugh score of A, and tumor size over 10 cm. Second, certain clinical data of treatment are not collected, including perioperative chemoradiotherapy, detailed surgical techniques, and usage of opioids during surgery. Opioids have been reported to have an effect on tumor cell proliferation and angiogenesis, as well as on tumor recurrence and metastasis. However, it’s hard to accurately record and compare total amounts of opioid used in both groups during surgery, as they were administered both continuously or intermittently. In this study all patients accepted at least one of remifentanil or sufentanil treatment in standard dose. Besides, since this is a retrospective analysis based clinical records and follow up data, the reason for the choice of anesthesia type at that time point, as well as potential factors affecting this choice, are not recorded or balanced. Thus, prospective researches with rigorous study design and large sample size on this field are in urgent need.

In conclusion, this retrospective analysis of long-term outcomes identifies that INHA is associated with worse survival rate compare with TIVA, and the choice of anesthesia type might be an independent risk factor for survival of HCC patients with PVTT. For some sub-variable groups (including PVTT type I, Child-puge B&C, tumor diameter < 10 cm, AFP <400μg/L) there was no difference in outcomes between TIVA and INHA. Future prospective researches are urgent to verify this difference and figure out underlying causes of it.

## Supplementary information

**Additional file 1: Table S1.** Cox Proportional Hazard Regression Analyses: Univariate Model for OS and RFS.

**Additional file 2.**

## Data Availability

The datasets used and/or analyzed during the current study are available from the corresponding author (WF Yu; ywf808@sohu.com) on reasonable request.

## Abbreviations

HCC: Hepatocellular carcinoma
PVTT: Portal vein tumor thrombus
INHA: Volatile inhalational anesthesia
TIVA: Total IV anesthesia
OS: Overall survival
MST: Median survival time
HIFs: Hypoxia-inducible factors
IGF: Insulin-like growth factor
MACE: Major adverse cardiac events
MOD: Multiple organ dysfunction
ALT: Alanine aminotransferase
AST: Aspartate aminotransferase
NLR: Neutrophil-lymphocyte ratio
ASA: American Society of anesthesiologists’ Assessment
AFP: Alpha-fetoprotein
