# Supplementary material for: Distant survival for patients undergoing surgery using volatile versus IV anesthesia for hepatocellular carcinoma with portal vein tumor thrombus: a retrospective study
Source: BMC Anesthesiol. 2020 Sep 14;20:233. doi: 10.1186/s12871-020-01111-w (PMC7491163; doi:10.1186/s12871-020-01111-w)
Supplement: Supplementary file 1 — Additional file 1: Table S1. Cox Proportional Hazard Regression Analyses: Univariate Model for OS and RFS. [file 12871_2020_1111_MOESM1_ESM.docx]

Supplemental table 1. Cox Proportional Hazard Regression Analyses: Univariate Model for OS and RFS.

|  | OS | |  | RFS | |
| --- | --- | --- | --- | --- | --- |
| Variables | HR (95% CI for HR) | *P* Value |  | HR (95% CI for HR) | *P* Value |
| Anesthesia type  (INHA/TIVA) | 1.287(1.063,1.559) | 0.010 |  | 1.223(1.014,1.476) | 0.035 |
| Age (continuous) | 0.997(0.987,1.006) | 0.460 |  | 0.993(0.984,1.003) | 0.158 |
| Sex (Female/Male) | 1.006(0.727,1.338) | 0.971 |  | 1.028(0.751,1.409) | 0.862 |
| ASA | 2.083(1.583,2.740) | <0.001 |  | 1.705(1.274,2.283) | <0.001 |
| Child-Pugh | 1.976(1.473,2.650) | <0.001 |  | 1.666(1.233,2.253) | 0.001 |
| AFP (ug/L) | 1.505(1.084,2.089) | 0.015 |  | 1.372(1.002,1.881) | 0.049 |
| Tumor Diameter (cm) | 1.74(1.283,2.346) | <0.000 |  | 1.55(1.171,2.051) | 0.002 |
| PVTT | 1.571(1.125,2.193) | 0.008 |  | 1.540(1.114,2.129) | 0.009 |
| WBC | 1.089(0.988,1.202) | 0.087 |  | 1.054(0.962,1.154) | 0.260 |
| ALT | 1.419(0.996,1.901) | 0.336 |  | 1.430(0.998,1.872) | 0.155 |
| AST | 1.720(0.998,2.122) | 0.060 |  | 1.804(0.895,2.483) | 0.096 |
| HBsAg-+ | 0.819(0.618,1.084) | 0.162 |  | 0.826(0.626,1.090) | 0.177 |
| Year of surgery | 0.936(0.904,1.025) | 0.232 |  | 0.944(0.889,1.003) | 0.063 |

* Overall categories comparison.

ASA = American Society of Anesthesiologists; PVTT=Portal vein tumor thrombus; INHA=Volatile inhalational anesthesia; TIVA=Total IV anesthesia., WBC=White blood cells, NLR=Neutrophil–lymphocytes ratio; SE=Standard Error, PSM=[Propensity Score Matching](http://www.baidu.com/link?url=vWyZXrXD6Qn2wed-2aJSHOkeEUN9KNsFfAQRUJAdg6CWo01ghtdmoOMItS8Zmf8XcdmQ5pq4gSvoqINuE0YrQK).
