# Supplementary figures and images for: Distant survival for patients undergoing surgery using volatile versus IV anesthesia for hepatocellular carcinoma with portal vein tumor thrombus: a retrospective study
Source: BMC Anesthesiol. 2020 Sep 14;20:233. doi: 10.1186/s12871-020-01111-w (PMC7491163; doi:10.1186/s12871-020-01111-w)

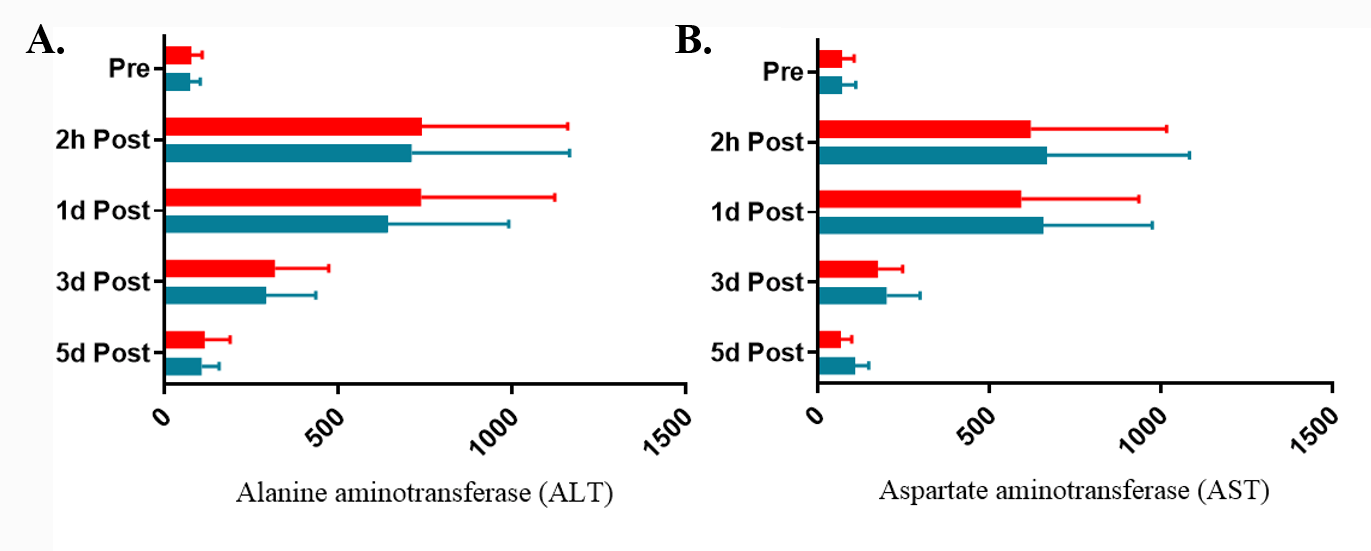

Supplement: Supplementary file 2 — Additional file 2. [file 12871_2020_1111_MOESM2_ESM.tif]
